# Smartphone Color Vision Testing as an Alternative to the Conventional Ishihara Booklet

**DOI:** 10.7759/cureus.30747

**Published:** 2022-10-27

**Authors:** Muhammad A Khizer, Umer Ijaz, Taimoor A Khan, Summaya Khan, Talha Liaqat, Abdullah Jamal, Izza Zahid, Hira G Shah, Muhammad A Zahid

**Affiliations:** 1 Ophthalmology, National University of Medical Sciences, Rawalpindi, PAK; 2 Ophthalmology, Armed Forces Institute of Ophthalmology, Rawalpindi, PAK; 3 Ophthalmology, National University of Medical Sciences, Quetta, PAK; 4 Medicine, Army Medical College, National University of Medical Sciences (NUMS), Rawalpindi, PAK; 5 General Medicine, National University of Medical Sciences, Rawalpindi, PAK; 6 Anaesthesiology, James Cook University, Townsville, AUS; 7 Ophthalmology, Alshifa Trust Eye Hospital, Rawalpindi, PAK; 8 Emergency Medicine, Casey Hospital, Monash Health, Berwick, AUS

**Keywords:** iphone, android, screen, color vision deficiency, smartphone, ishihara plates

## Abstract

Introduction

Color vision testing was first seen as a parameter to be tested in the 1700s. Nowadays, it is a well-known phenomenon with significant quality-of-life implications. Structures involved in color vision include the lens, pupil, retinal cone photopigments, and several photoreceptor processes that translate the incoming spectrum of different light wavelengths into a processed colored image. An initial color vision assessment was made simply by comparing the color perception of the individual to that of the examiner. The most commonly used tools to screen for color vision defects today are color plates, such as the Ishihara color plates. In the modern age, smartphones have evolved to become an essential part of our everyday lives with applications such as Eye Handbook, which allow easier access to color vision testing using color plates displayed on smartphone screens. In this study, we compared color vision testing on Android and iOS devices to the standard Ishihara booklet.

Materials and methods

A cross-sectional validation study was performed on patients presenting to the Armed Forces Institute of Ophthalmology, Rawalpindi, Pakistan for six months. The sample size collected was 162 with a 95% confidence interval. The age range of the sample population was kept at 12-70 years. A patient was selected for participation in the study, and a color vision assessment was performed using the Ishihara color plates and Android and iOS smartphones. The collected data was then entered into IBM SPSS (Statistical Package for the Social Sciences) Statistics 25 for analysis, with the p-value being kept at 0.05.

Results

The sample size was 162, with the gender distribution being predominantly male (69.14%). The average age of the participants was 35.94 (*SD* = 12.04). The result of the two-tailed paired sample z-testwas not significant based on a p-value of 0.565, indicating the null hypothesis cannot be rejected. This finding suggests the difference between the mean of Ishihara and the mean of the iPhone was not significantly different from zero. Similar results were found for comparisons between Android smartphones and the Ishihara booklet.

Conclusions

Previous studies conducted showed nearly 60% of subjects with normal color vision correctly identified all colors on standard Ishihara color plates. The two-tailed paired sample t-test conducted in our study showed no significant difference between either of the smartphone groups (iPhone or Android) and the Ishihara booklet group, indicating that smartphones present a viable alternative to standard Ishihara booklet testing. However, there are certain limitations to our study. Different types of smartphone screens present a challenge in standardization while testing color vision, something that is not a problem when using the Ishihara booklet. However, smartphones are more widely available, more versatile, and present far greater ease of access. Both these factors should be considered when comparing the two in future studies.

## Introduction

Color vision testing has come a long way from the time it was first seen as a parameter to be tested in the 1700s to today when it is a well-known phenomenon with color vision defects having real-life implications affecting the quality of life of an individual. Color vision theory describes the mechanism of color vision and proposes several structures involved in the processing of the incoming light to produce the phenomenon of color. Structures involved include the lens, pupil, and retina for image reception and cone photopigments, and several photoreceptor processes which translate the incoming spectrum of different light wavelengths into a processed colored image [1]. Any defect in this mechanism results in alterations in normal color perception. In a study conducted in Manipur, India, in a study group of six different populations, about 8.73% of males and 1.69% of females were found to have color vision defects [2]. Similar findings were observed in a study conducted among various ethnic groups of students in Erbil city, where 8.47% of males and 1.37% of females were found to be color blind [3].

The initial assessment of color vision was made simply by comparing the color perception of the individual being tested to that of the examiner. However, with time, as the understanding of color vision improved, more sophisticated tests began to be used. The pseudo-isochromatic plates developed by the German scientist J. Stilling [4] were one of these. Today, the most commonly used tools to screen for color vision defects are color plates, with some of the most prevalent being Ishihara color plates and H-R-R polychromatic plates [5]. Color vision testing is regularly performed nowadays as a part of routine ophthalmologic screening.

While the Ishihara and the Hardy-Rand-Rittler (HRR) color tests provide standardized and well-studied testing tools for color vision assessment, limitations such as availability, image quality, and aging of the color plates with time may affect their usefulness [5]. In the modern age, smartphones have evolved to become an essential part of our everyday lives. The estimated number of smartphone users in the world in 2021 was around 6.259 billion, with this number being projected to grow to 7.690 billion by 2027 [6]. Due to the widespread use of smartphones, tools have been developed that allow clinicians to implement them even in a professional setting. One such tool is the development of applications that can allow color vision assessment. These applications may contain one of the many color vision assessment plates, allowing more versatility and, due to the widespread usage of smartphones, much greater ease of access. Of these applications, Eye Handbook (EHB) is mentioned in the literature as a viable digital alternative to the printed Ishihara booklet for screening purposes [5,7,8].

A major limitation of these studies is that they are tested only on iOS smartphones, whereas Android smartphones are vastly more widespread in use. Moreover, the same application used on another platform may perform differently despite being marketed as being the same [5,7,9,10]. For example, the majority of published literature included only subjects who had congenital color vision defects, whereas literature mentions the usage of the Ishihara test for conditions associated with acquired color vision defects (for example, optic neuropathies and macular disorders) [11,12].

To the best of our knowledge, the iOS version of EHB has not been compared with its Android version, and only Ozgur et al included a subset of patients with ocular disorders in their study [13]. In this study, we evaluate color vision testing by a smartphone application on both iOS and Android devices, and along with comparing them, we also include patients with concurrent ocular disorders.

## Materials and methods

A cross-sectional study was performed on patients presenting to the Armed Forces Institute of Ophthalmology, Rawalpindi, Pakistan. The study was conducted for six months, with the sample size being calculated using the OpenEpi online sample size calculator [14]. The sample size collected was 162 with a 95% confidence interval, 11% population proportion, and 5% precision. Sample collection was done via convenience sampling. The ethical review board of the Armed Forces Institute of Ophthalmology, Rawalpindi, provided the approval to conduct the study (certification number 212/ERC/AFIO dated February 14, 2020).

An inclusion criterion was defined with the patients selected for testing being those with a documented color vision defect on standard Ishihara booklet testing and best corrected visual acuity of at least 6/15 in the better eye (diseased group). The age range of the sample population was kept at 12-70 years, and they were all able to correctly read numbers. Individuals with central nervous system pathology (for example, multiple sclerosis, and giant cell arteritis) that affected their vision were excluded from the study.

A basic data collection instrument was used to gather demographic details, biodata, ophthalmic data, and information about the diseased condition. Data were collected from patients after obtaining informed consent and adequate information regarding the reason for collection. Patient anonymity was maintained at all times, with only the researchers having access to patient information. At no point was this data shared or distributed to a third party.

A patient was selected for participation in the study, and a color vision assessment was performed using the Ishihara color plates. After that, color vision assessment was performed using the Ishihara plates on Eye Handbook (a smartphone application by Cloud Nine Development LLA®-Copyright 2009-2022) application on both iOS (by Apple Developer, installed on Apple iPhone 11 Pro Smartphone) and Android OS (by Google Alphabet Inc, installed on Samsung S-21 Smartphone) separately. Both results were attached to the primary data collection instrument for further analysis. The collected data was then entered into IBM SPSS 25 for analysis [15]. The frequencies of age and sex of participants were also calculated to assess any statistically significant correlations. For the reliability coefficient to accurately reflect the true reliability of test results, a two-scale Cronbach’s coefficient alpha (Scale 1 and 2) was applied along with a two-tailed paired sample z-test for Pearson correlation between color vision test results from Ishihara, iOS, and Android EHB versions. A p-value of less than 0.05 was considered statistically significant.

## Results

The sample size was 162, with the gender distribution being predominantly male (69.14%). All of the participants completed the study with none of them dropping out. The sample population was also analyzed for the presence of any ocular pathology. Ocular pathology was present in 22.22% of the participants, with optic neuropathy being the most common (9.88%). Other diagnosed pathologies include retinal disease, cataracts, and corneal disease. The average age of the participants was 35.94 (SD = 12.04). Gender distribution and the presence or absence of eye pathology are shown in Table 1.

**Table 1:** Frequency table for nominal variables

Variable	n	%
Gender		
Male	112	69.14
Female	50	30.86
Missing	0	0.00
Eye		
Right	81	50.00
Left	81	50.00
Missing	0	0.00
Group		
Non-diseased	126	77.78
Diseased	36	22.22
Missing	0	0.00
DiseaseType		
Non-diseased	124	76.54
Optic neuropathy	16	9.88
Retinal disease	10	6.17
Cataract	5	3.09
Corneal disease	7	4.32

A Cronbach alpha coefficient was calculated for scale 1, consisting of serial, age, Ishihara, Android, and iPhone. The Cronbach's alpha coefficient was evaluated using the guidelines suggested by George and Mallery (2018) where >.9 is excellent, > .8 good, > .7 acceptable, > .6 questionable, > .5 poor, and ≤ .5 unacceptable [16].

The items for one had a Cronbach's alpha coefficient of .32, indicating unacceptable reliability. The following variables were negatively correlated with the overall composite score: serial. These variables were automatically reverse-coded to improve reliability. Table 2 presents the results of the reliability analysis.

**Table 2:** Reliability table for scale 1 - Cronbach's alpha

Scale	No. of Items	α	Lower bound	Upper bound
1	5	.32	.23	.40
Note. The lower and upper bounds of Cronbach's α were calculated using a 95.00% confidence interval.				

A Cronbach alpha coefficient was calculated for scale 2, consisting of Ishihara, Android, and iPhone. The Cronbach's alpha coefficient was evaluated using the guidelines suggested by George and Mallery (2018) where >.9 is excellent, > .8 good, > .7 acceptable, > .6 questionable, > .5 poor, and ≤ .5 unacceptable [16].

The items for two had a Cronbach's alpha coefficient of 1.00, indicating excellent reliability. Table 3 presents the results of the reliability analysis.

**Table 3:** Reliability table for scale 2 - Cronbach's alpha

Scale	No. of Items	α	Lower bound	Upper bound
2	3	1.00	1.00	1.00
Note. The lower and upper bounds of Cronbach's α were calculated using a 95.00% confidence interval.				

A two-tailed paired sample z-test was conducted to examine whether the mean difference between Ishihara and iPhone was significantly different from zero.

**Figure 1:**
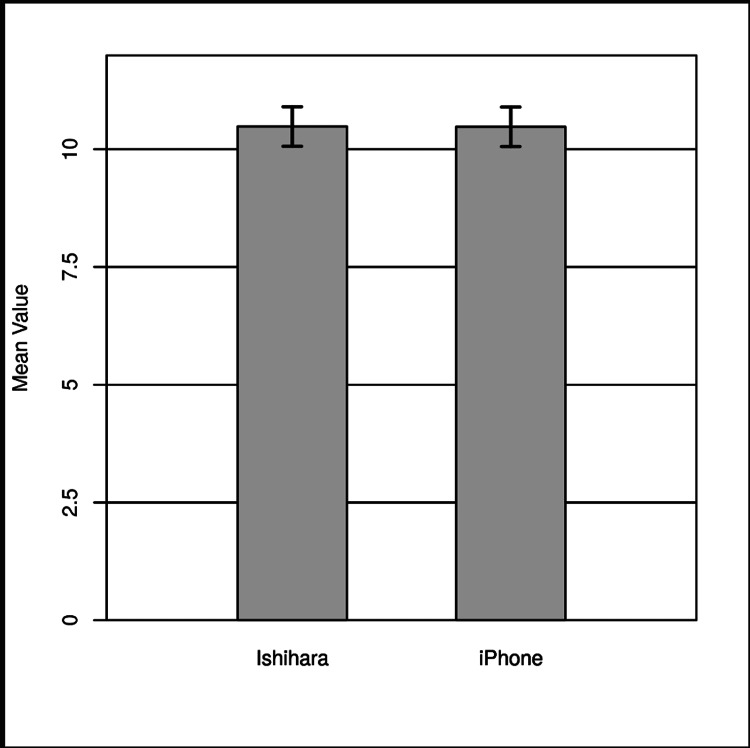
Two-tailed paired sample z-test for the difference between Ishihara and iPhone

The result of the two-tailed paired sample z-test was not significant based on an alpha value of .05, z = 0.58, p =.565, indicating the null hypothesis cannot be rejected. This finding suggests the difference between the mean of Ishihara and the mean of the iPhone was not significantly different from zero. The results are presented in Table 4 and Figure 1.

**Table 4:** Two-tailed paired sample z-test for the difference between Ishihara and iPhone

Ishihara		iPhone			
M	SD	M	SD	z	p
10.48	2.73	10.48	2.72	0.58	.565
Note. N = 162.					

A two-tailed paired sample t-test was conducted to examine whether the mean difference between Ishihara and Android was significantly different from zero.

**Figure 2:**
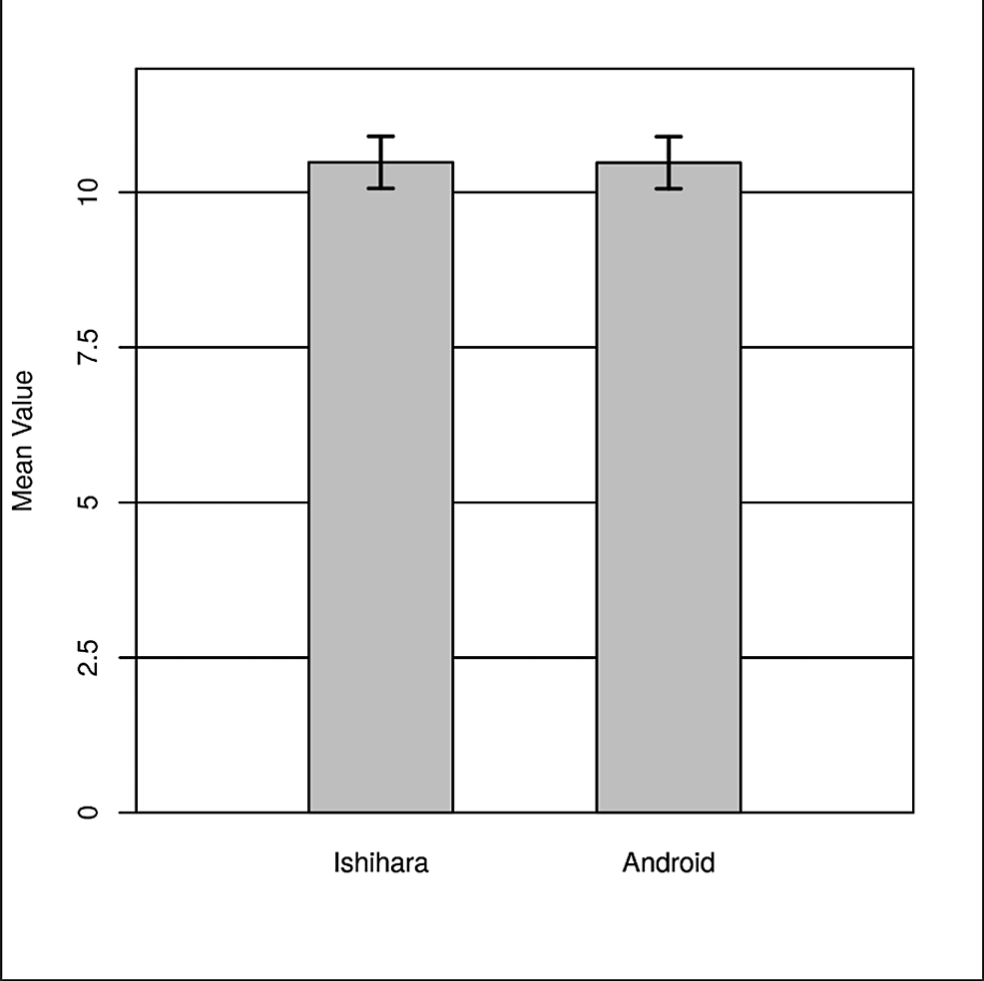
Two-tailed paired sample t-test for the difference between Ishihara and Android

The result of the two-tailed paired samples t-test was not significant based on an alpha value of .05, t (161) = 0.58, p = .565, indicating the null hypothesis cannot be rejected. This finding suggests the difference between the mean of Ishihara and the mean of Android was not significantly different from zero. The results are presented in Table 5 and Figure 2.

**Table 5:** Two-tailed paired sample t-test for the difference between Ishihara and Android

Ishihara		Android				
M	SD	M	SD	t	p	d
10.48	2.73	10.48	2.72	0.58	.565	0.05
Note. N = 162. Degrees of freedom for the t-statistic = 161. d represents Cohen's d.						

A Friedman rank sum test was conducted to examine whether the medians of Ishihara, Android, and iPhone were equal. The Friedman test is a non-parametric alternative to the repeated measures one-way ANOVA and does not share the ANOVA's distributional assumptions [17,18].

**Figure 3:**
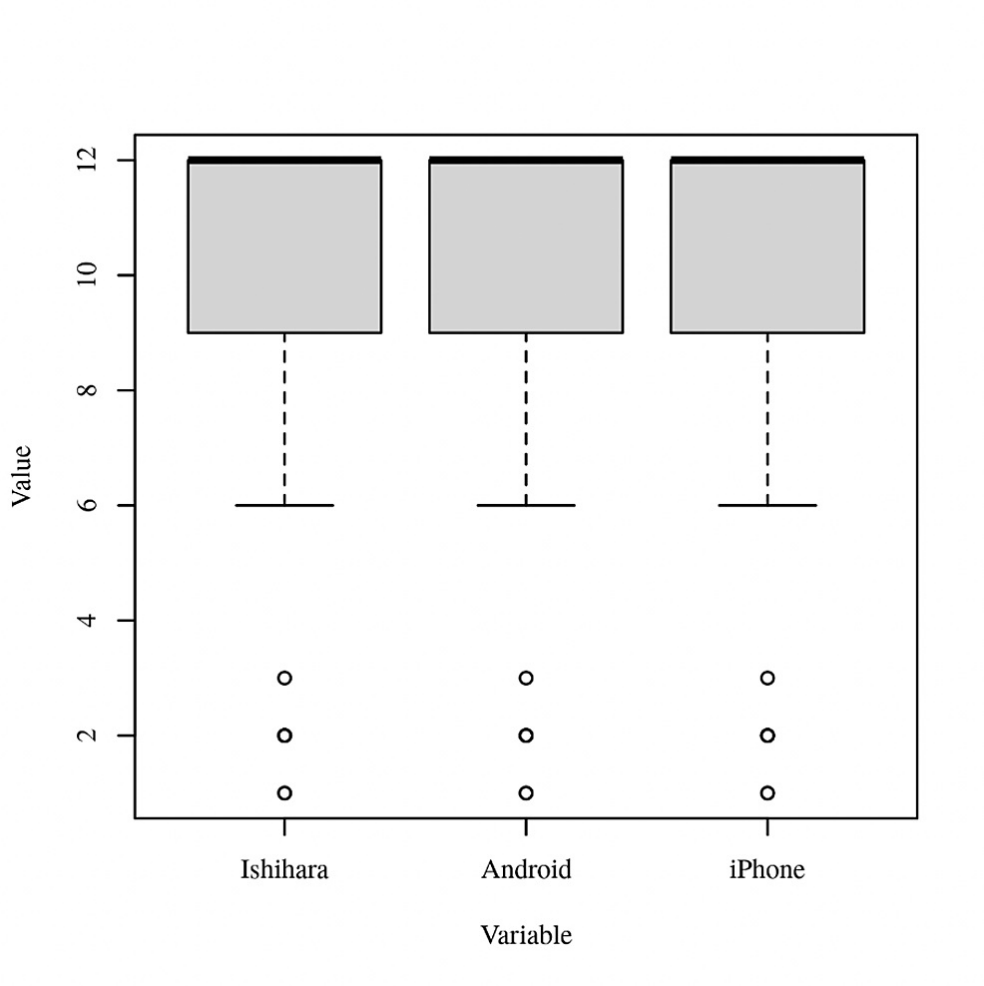
Boxplots of Ishihara, Android, and iPhone

The results of the Friedman test were not significant based on an alpha value of .05, χ2(2) = 0.67, p =.717, indicating no significant differences in the median values of Ishihara, Android, and iPhone. Table 6 presents the results of the Friedman rank sum test. Figure 3 presents boxplots of Ishihara, Android, and iPhone.

**Table 6:** Friedman rank sum test

Variable	Mean rank	χ^2^	df	p
Ishihara	2.01	0.67	2	.717
Android	2.00			
iPhone	2.00			

## Discussion

Previous studies conducted showed nearly 60% of subjects with normal color vision correctly identified all colors on standard Ishihara color plates. About 40% of individuals with normal color vision made at least one misreading, but none of them made any errors, which might indicate a red-green color deficiency [19]. In a study comparing the standard Ishihara color plates to those available in the EHB application, 78% of participants correctly identified the plates in the Ishihara color plate group while 85% correctly identified the plates in the EHB group [13]. A two-tailed paired sample t-test conducted in our study showed no significant difference between either of the smartphone groups (iPhone or Android) and the Ishihara booklet group.

A study conducted in 2012 comparing the Ishihara booklet to EHB in a sample of 100 random male patients showed no significant differences between either group in correctly identifying the color plates. The iPhone diagnosed color defects with the same efficacy as the standard booklet [20]. Our study, contrary to previous ones, compared both iOS and Android versions of the EHB to the Ishihara plates and showed that not only is there no statistically significant difference between the smartphone group and the Ishihara booklet group, but the p-value for comparisons between Android phones and iPhones is also >0.05, thus rejecting the null hypothesis and showing that both iPhone and Android phones have similar efficacy in diagnosing color vision defects.

A study conducted by Filotsos MJ et al. also compared this popular application with the Ishihara booklet in normal individuals. They highlighted that contrast-related effects are also an individual factor that has not been standardized in the EHB application and may have altered the color vision (CV) test outcome [21]. Furthermore, Anandanam A et al. have documented that the CV significantly decreases with a decrease in contrast sensitivity. Thus, these independent variables must be taken into consideration when the EHB application is standardized and approved as an alternate and validated CV testing modality [22].

Limitations

There are certain limitations to our study. The fact that smartphone screens have significant differences between them is not to be understated. There is a large variety of smartphone screens currently available, ranging from Liquid Crystal Display panels to Organic Light Emitting Diode, Light Emitting Diode, and micro-LED displays. These different types of displays present a challenge to standardization as compared to the Ishihara booklet, which has an established standard of quality and visibility. Likewise, during testing, the difference in brightness of the smartphone screen display may affect the visibility of the color plates in the EHB application. While brightness can be controlled in the case of the Ishihara booklet by testing in similar lighting conditions, in the case of smartphone applications, different smartphone screens have different brightness settings, and this might present as a potential confounder in testing color vision. Differences in smartphone screen type and brightness were not taken into account and may affect the results obtained.

## Conclusions

Although color vision testing on smartphones presents itself as a convenient and more widely available alternative to Ishihara color plate booklets, the lack of standardization between different smartphone screens is a point of concern. In addition, the current landscape of the smartphone industry is rapidly changing, with better, brighter, and higher-resolution screens constantly being developed. It is difficult to assess every individual screen as the variety of smartphones in the current market is vast and testing them all for standardization presents a significant challenge. In regards to standardization, Ishihara color plate booklets present a better alternative to smartphone screens. However, smartphones are more widely available, more versatile, and present far greater ease of access. Both these factors should be considered when comparing the two in future studies.
